# Effect of treatment with epoetin-*β* on survival, tumour progression and thromboembolic events in patients with cancer: an updated meta-analysis of 12 randomised controlled studies including 2301 patients

**DOI:** 10.1038/sj.bjc.6604408

**Published:** 2008-06-10

**Authors:** M Aapro, A Scherhag, H U Burger

**Affiliations:** 1 Institut Multidisciplinaire d'Oncologie, Clinique de Genolier, 1, route du Muids, Genolier CH-1272, Switzerland; 2 F Hoffmann-La Roche Ltd, Basel CH-4070, Switzerland

**Keywords:** anaemia, epoetin-*β*, survival

## Abstract

Epoetin-*β* is used to treat patients with metastatic cancer undergoing chemotherapy to alleviate the symptoms of anaemia, reduce the risk of blood transfusions and improve quality of life. This meta-analysis of 12 randomised, controlled studies evaluated the impact of epoetin-*β* on overall survival, tumour progression and thromboembolic events (TEEs). A total of 2297 patients were included in the analysis (epoetin-*β*, *n*=1244; control, *n*=1053; 65% solid and 35% nonmyeloid haematological malignancies). A prespecified subgroup analysis assessed the effects in patients with a baseline Hb⩽11 g dl^−1^, corresponding to current European Organisation for Research and Treatment of Cancer (EORTC) guidelines. No statistically significant effect on mortality was observed with epoetin-*β vs* control, both overall (hazard ratio (HR)=1.13; 95% CI: 0.87, 1.46; *P*=0.355) and in patients with baseline Hb⩽11 g dl^−1^ (HR=1.09; 95% CI: 0.80, 1.47; *P*=0.579). A trend for a beneficial effect on tumour progression was seen overall (HR=0.85; 95% CI: 0.72, 1.01; *P*=0.072) and in patients with an Hb⩽11 g dl^−1^ (HR=0.80; 95% CI: 0.65, 0.99; *P*=0.041). An increased frequency of TEEs was seen with epoetin-*β vs* control (7 *vs* 4% of patients); however, TEEs-related mortality was similar in both groups (1% each). The results of this meta-analysis indicate that when used within current EORTC treatment guidelines, epoetin-*β* has no negative impact on survival, tumour progression or TEEs-related mortality.

Anaemia is a common occurrence in patients with cancer, arising either as a result of the underlying malignant disease, as a consequence of myelosuppressive chemotherapy or radiotherapy, or a combination of both (Bokemeyer *et al*, 2005). Anaemia is associated with a multitude of symptoms that have a profound impact on a patient's condition and quality of life (QoL) (Ludwig *et al*, 2004). Furthermore, as an independent prognostic factor, anaemia has consistently been shown to be associated with adverse outcomes in patients with a variety of malignancies (Caro *et al*, 2001).

Erythropoiesis stimulating agents (ESAs) have been shown to increase Hb levels and reduce transfusion requirements in patients with cancer (Littlewood *et al*, 2001; Österborg *et al*, 2002; Vansteenkiste *et al*, 2002). In addition, treatment with ESAs has been shown to alleviate fatigue, one of the most distressing symptoms of anaemia, and result in an improvement in patients' QoL when compared with placebo or standard transfusion therapy (Littlewood *et al*, 2001; Crawford *et al*, 2002).

Preclinical data have suggested an enhanced tumour response and delayed tumour progression associated with ESA treatment (Mittelman *et al*, 2001; Thews *et al*, 2001; Stuben *et al*, 2003). Moreover, in clinical studies, a potential survival benefit has been shown in patients undergoing cancer therapy who received treatment with ESAs (Antonadou *et al*, 2001; Glaser *et al*, 2001; Littlewood *et al*, 2001). The results of the first meta-analysis of 19 randomised, controlled trials in 2865 cancer patients receiving ESAs, reported by the Cochrane Group, showed a trend towards increased survival in patients treated with ESAs (hazard ratio (HR)=0.81; 95% CI: 0.67, 0.99; *P*=0.04) and lent support to these findings (Bohlius *et al*, 2005).

In contrast to the above, two studies, one in patients with head and neck cancer (Henke *et al*, 2003) and the other in patients with breast cancer (Leyland-Jones *et al*, 2005) found higher rates of tumour progression and decreased survival, respectively, in patients receiving ESA treatment compared with placebo. Both studies had a number of methodologic limitations, including baseline imbalances and protocol violations, which confounded the interpretation (Leyland-Jones and Mahmud, 2004; Vaupel and Mayer, 2004). However, the results of an updated Cochrane meta-analysis by Bohlius *et al* (2006) of 57 trials including 9353 patients also found a shift of the HR for survival (HR=1.08; 95% CI: 0.99, 1.18; *P*=0.10) towards an increased risk for patients receiving various ESAs.

We previously reported the results of a meta-analysis of nine randomised, controlled studies of epoetin-*β* conducted in 1403 patients undergoing cancer therapy (Aapro *et al*, 2006). This meta-analysis did not demonstrate any evidence that treatment with epoetin-*β* impairs survival (HR=0.97; 95% CI: 0.69, 1.36; *P*=0.87) or promotes tumour progression (HR=0.78; 95% CI: 0.62, 0.99; *P*=0.042). However, one limitation of these findings was the short duration of follow-up (28 days), particularly for survival.

The present update to this previous meta-analysis reports the results from 12 randomised, controlled studies with epoetin-*β* in 2301 patients receiving anticancer therapy, including three recently completed trials with longer term follow-up in patients with head and neck cancer (Henke *et al*, 2003), patients with metastatic breast cancer (Aapro *et al*, 2008) and patients with cervical cancer (Strauss *et al*, 2008).

## Materials and methods

This updated meta-analysis, using individual patient data, was designed to evaluate differences between epoetin-*β* and control (placebo or standard care) with regard to overall survival, disease progression and thromboembolic events (TEEs) during and up to 28 days after end of therapy with epoetin-*β*. Eligible studies included all randomised, controlled studies of epoetin-*β* conducted by the drug sponsor (F Hoffmann-La Roche or Boehringer Mannheim) in patients with cancer undergoing treatment (chemotherapy (seven studies), surgery (two studies), radiotherapy (two studies) or radio-chemotherapy (one study)). A subgroup analysis of four controlled studies with extended follow-up provides information on the effect of epoetin-*β* on long-term survival and malignancy progression. Individual study details are summarised in Table 1.

The primary objectives of the meta-analysis were to evaluate the effects of epoetin-*β* on overall survival, disease progression and TEEs in cancer patients in the overall data set, and for solid tumours and nonmyeloid haematological malignancies separately. Secondary predefined objectives included the evaluation of the potential impact of various Hb intervention levels on mortality and disease progression as well as evaluation of the latter outcomes in a predefined subgroup analysis including patients with long-term follow-up or those with an Hb level at baseline (intervention Hb)⩽11 g dl^−1^.

Most of the studies were originally designed to evaluate the efficacy of epoetin-*β* with respect to anaemia correction and thus there was no follow-up for survival or tumour progression beyond study treatment plus a standard 28-day period used to assess SAEs, including deaths and disease progression. Although tumour status was not prospectively assessed in many of the earlier trials with short-term follow-up, details of disease progression were routinely reported as adverse events. For the present meta-analysis, this information was analysed retrospectively by reviewers blinded to treatment assignment. Other studies were, however, designed to assess the effects of epoetin-*β* on survival and/or disease progression (Henke *et al*, 2003; Aapro *et al*, 2008) or Hb response to treatment (Strauss *et al*, 2008). Long-term follow-up information, up to 60 months, was available for overall survival in four studies (Henke *et al*, 2003; Österborg *et al*, 2005; Aapro *et al*, 2008; Strauss *et al*, 2008) and for tumour progression in three studies (Henke *et al*, 2003; Aapro *et al*, 2008; Strauss *et al*, 2008). All reported adverse events were also reviewed against a prespecified list of TEEs, the definition of which was consistently applied across all studies.

### Statistical analyses

Overall survival and time to progression were analysed by Kaplan–Meier estimates, log-rank testing and Cox regression analysis. Thromboembolic events were summarised in terms of crude rates independent of onset. Time to TEEs was analysed as for survival and time to progression. Differences in duration of survival, time to tumour progression and time to TEEs (time between start of epoetin/control therapy or baseline visit and time of event) were tested using log-rank tests.

Two sets of analyses were performed. One analysis included data from all 12 studies. For these analyses, patients without events were censored at 4 weeks after the last entry in the drug administration record. A second analysis using only pooled data from the studies with long-term follow-up, in which all events were included in the analysis, was performed for overall survival (all four studies) and time to progression (three studies). In the study by Österborg *et al* (2005), patients were followed for survival but not for disease progression; therefore; this study was excluded from the time-to-progression analyses. Patients without an event were censored at the time of last follow-up or, if no follow-up information was available, 4 weeks after the last entry in the administration record.

A predefined subgroup analysis was performed using a subgroup of patients with a baseline Hb intervention level corresponding to the European Organisation for Research and Treatment of Cancer (EORTC) guidelines (i.e., ⩽11 g dl^−1^).

The primary analysis was based on a simple pooling strategy without further stratification. Analyses stratified by study were also performed.

## Results

### Analysis populations

A total of 2301 patients were enrolled in the 12 trials of whom 2297 (epoetin-*β*, *n*=1244; control, *n*=1053) were included in the analysis; four patients who received no treatment of any kind during the trials were excluded. All patients who received at least one dose of study medication were analysed according to the treatment received. Five patients randomised to control received epoetin-*β* and three patients in the epoetin group received no epoetin-*β* treatment.

### Baseline characteristics and follow-up

Of the 2297 patients in the analysis, 35% had nonmyeloid haematological malignancies and 65% had solid tumours (Table 2). Most patients with solid tumours had primary malignancies of the breast, head and neck, colon/rectum and ovary. Among patients with nonmyeloid haematological malignancies, the most common were non-Hodgkin's lymphoma (56%) and multiple myeloma (41%). A slightly higher proportion of patients in the epoetin group had ovarian carcinoma as a result of the three arm design of the study by ten Bokkel Huinink *et al* (1998). No other clinically relevant differences between the groups were noted. Tumour stage at baseline was not consistently collected in the various studies as assessment of tumour progression was not a predefined study objective in most of the earlier studies. However, information on tumour stage (FIGO or TNM) was available for nearly 70% of patients with solid tumours. There were no relevant differences between the treatment arms with respect to tumour staging with the exception of FIGO Stage III, which was more common in the epoetin-*β* arm (16%) than in the control arm (8%) of patients with solid tumours in which this staging criteria was used.

Mean baseline Hb level was 10.6 g dl^−1^ in the control arm and 10.5 g dl^−1^ in the epoetin-*β* arm. Median initial weekly epoetin-*β* dose was 27 000 IU (range 0–90 000 IU). During treatment, mean maximum Hb level was 12.0 g dl^−1^ in the control arm and 13.4 g dl^−1^ in the epoetin-*β* arm. The mean baseline-adjusted Hb area under the curve was 0.07 g dl^−1^ with control compared with 1.24 g dl^−1^ with epoetin-*β*.

Duration of follow-up across the 12 studies was comparable in the control (median 3.8 months) and epoetin-*β* (median 3.9 months) treatment groups (patients without events from the four studies with long-term follow-up were censored 4 weeks after last entry in the administration record). In the four studies with long-term follow-up data, when all events were included, median follow-up was also comparable (29.8 months with control and 28.8 months with epoetin-*β*).

### Effects on survival

**Figure 1:**
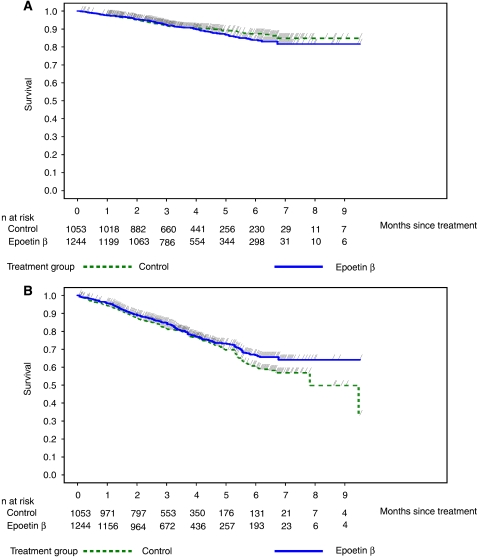
Kaplan–Meier curves of (**A**) overall survival and (**B**) time to progression in the pooled population of 12 controlled studies.

The death rate in the control group was 0.29 deaths per patient-year and 0.33 in the epoetin-*β* group (Table 3). There was no statistically significant difference between patients receiving epoetin-*β* or control (standard treatment) in terms of overall survival in the pooled analysis of all 12 controlled studies (data collected up to 28 days after last dose) (HR=of 1.13; 95% CI: 0.87, 1.46; log-rank, *P*=0.355) (Figure 1A). Time-to-event analyses, however, suggested a numerically increased risk for mortality in the epoetin-*β* arm *vs* control with respective overall event rates of 10.9 and 9.4%. Comparable results were found in the pooled analysis of four studies with long-term follow-up. Mortality rates were 0.39 and 0.44 deaths per patient-year in the control and epoetin-*β* groups, respectively. Median survival was 20.6 months for control and 17.8 months for epoetin-*β*, with overall event rates of 60.5 and 64.5% and an HR of 1.13 (95% CI: 0.98, 1.31; log-rank, *P*=0.082) (Table 3). It is worth noting that the trend observed in this substudy analysis was due to the larger number of events associated with a longer follow-up.

In both the analysis of the pooled population of 12 controlled studies (including events up to 28 days after end of treatment) and the analysis of four studies with long-term follow-up, the risk of death for patients with solid tumours (HR=1.17; 95% CI: 0.83, 1.64 and HR=1.17; 95% CI: 0.99, 1.39, respectively) was similar to that in the overall pooled population (HR=1.13; 95% CI: 0.87, 1.46 and HR=1.13; 95 % CI: 0.98, 1.31, respectively), however, it was lower for patients with nonmyeloid haematological malignancies (HR=1.04; 95% CI: 0.69, 1.55 and HR=1.04; 95% CI: 0.80, 1.36, respectively) (Table 3).

**Figure 2:**
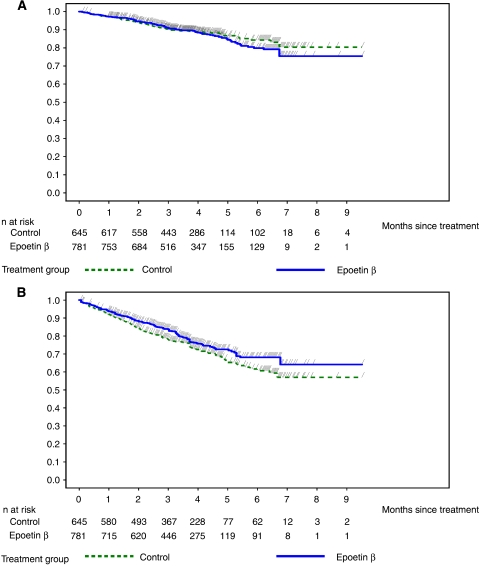
Kaplan–Meier curves of (**A**) overall survival and (**B**) time to progression in patients with a baseline Hb⩽11 g dl^−1^ in the pooled population of 12 controlled studies.

In the subgroup of patients with baseline Hb⩽11 g dl^−1^ from the pooled analysis of 12 controlled studies (*N*=1426) (i.e., in line with current EORTC guidelines), the overall event rate was comparable (11.5 and 12.5% for control and epoetin-*β*, respectively), and time-to-event analyses showed a HR of 1.09 (95% CI: 0.80, 1.47; log-rank, *P*=0.580) (Figure 2A). In the pooled population of four studies with long-term follow-up, the overall event rate was 68% for control and 70% for epoetin-*β*; median survival was 15.9 months in both treatment arms and the estimated HR=1.03 (95% CI: 0.85, 1.25; log-rank, *P*=0.750).

**Figure 3:**
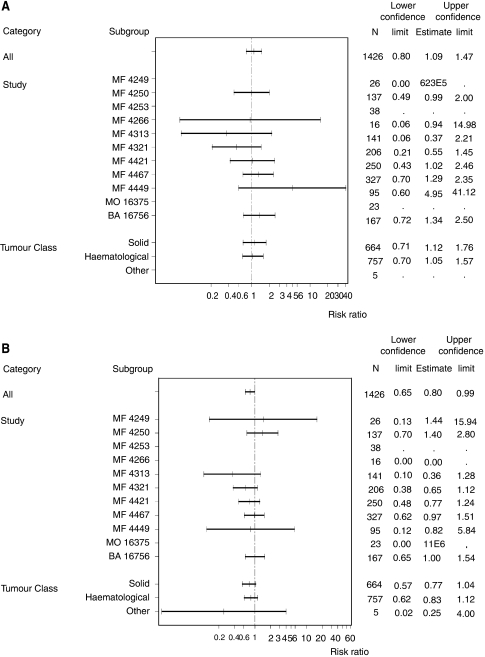
Summary of hazard ratios for (**A**) overall survival and (**B**) time to progression in subgroup of patients with baseline Hb⩽11 g dl^−1^.

No significant differences in overall survival were seen between epoetin-*β* and control in patients with solid tumours or nonmyeloid haematological malignancies in the subgroup of patients with baseline Hb⩽11 g dl^−1^ (Figure 3A).

### Effects on disease progression

No significant differences between the epoetin-*β* and control groups were seen in the number of patients with disease progression in the overall study population. The rates of disease progression were lower in patients receiving epoetin-*β* (0.74 events per patient-year) as compared to those in the control arm (0.86 events per patient-year)(Table 3). In the overall pooled population of the 12 controlled randomised studies, Kaplan–Meier analysis indicated a similar risk of progression, with a trend in favour of a reduced risk among patients treated with epoetin-*β* (HR=0.85; 95% CI: 0.72, 1.01; log-rank, *P*=0.072) (Figure 1B). In both subgroups of patients with solid or nonmyeloid haematological tumours, the HRs for disease progression were similar to that for the overall population (Table 3).

In the pooled analysis of the three studies where long-term disease progression follow-up was recorded, the rate of disease progression was higher (0.62 events per patient-year) compared with those in the control arm (0.54 events per patient-year). The overall event rate was 59% with control and 61% with epoetin-*β*, and the risk of progression was similar, with a trend for a higher risk of disease progression in patients receiving epoetin-*β* (HR=1.13; 95% CI: 0.95, 1.34; log-rank, *P*=0.165) (Table 3). Median time to progression was 11.2 months with control and 9.8 months with epoetin-*β*.

In the subgroup of patients with baseline Hb⩽11 g dl^−1^, in the pooled analysis of 12 controlled studies, the percentage of patients with disease progression was lower with epoetin-*β* (22.2%) than with control (27.0%). Similarly, in the pooled analysis of studies with long-term follow-up, a lower percentage of patients in the epoetin-*β* arm had disease progression (61.0%) compared with the control arm (69.4%). Time-to-event analyses showed a reduced risk of progression for patients with a baseline Hb⩽11 g dl^−1^ receiving epoetin-*β* in the pooled analysis of 12 controlled studies (HR=0.80; 95% CI: 0.65, 0.99; log-rank, *P*=0.041) (Figure 2B) and a trend towards a reduced risk with epoetin-*β* in the pooled analysis of three studies with long-term follow-up (HR=0.85; 95% CI: 0.64, 1.13; log-rank, *P*=0.267).

No significant differences in time to progression were seen between epoetin-*β* and control in patients with solid tumours or nonmyeloid haematological malignancies in the subgroup of patients with baseline Hb⩽11 g dl^−1^ (Figure 3B).

### Effects on thromboembolic events

Across the 12 studies in the pooled analysis, there is a statistically significantly shorter time to TEEs in the epoetin-*β* group compared with control (*P*=0.0075, log-rank test). Furthermore, a higher incidence of TEEs was seen with epoetin-*β* (7.1%) *vs* control (4.4%), largely due to reports of deep vein thrombosis (1.3 *vs* 0.4%), thrombophlebitis (0.6 *vs* 0.3%) and pulmonary embolism (1.2 *vs* 0.9%). However, there was no difference in the incidence of fatal TEEs between the treatment arms (1% each), the most common being pulmonary embolism.

The TEEs rate was higher in the epoetin-*β* group (0.22 events per patient-year) compared with the control (0.14 events per patient-year) with an overall HR for time to TEEs of 1.62 (95% CI: 1.13, 2.31; log-rank, *P*=0.008). The risk of TEEs in patients receiving epoetin-*β* was higher in the subgroup of patients with solid tumours (HR=1.92; 95% CI: 1.24, 2.99) than in those with nonmyeloid haematological malignancies (HR=1.18; 95% CI: 0.64, 2.16) (Table 3).

### Sensitivity analyses

Results from analyses adjusting by study yielded results consistent with the primary analysis (data not shown).

## Discussion

The results of the meta-analysis of 12 randomised controlled studies in patients with solid tumours or nonmyeloid haematological tumours (*n*=2297) treated with epoetin-*β* or control/placebo do not show any significant negative effect of epoetin-*β* on survival or tumour progression. Importantly, this updated meta-analysis also includes long-term follow-up data from more recent studies and largely confirms the results of the earlier meta-analysis of nine controlled studies (*n*=1413) (Aapro *et al*, 2006), which did not include the recently completed studies by Henke *et al* (2003); Aapro *et al* (2008) and Strauss *et al* (2008). Moreover, this update confirms the safety of epoetin-*β* in terms of overall survival and disease progression when used within current EORTC guidelines with respect to an intervention Hb level⩽11 g dl^−1^.

### Overall survival

The results for overall survival are consistent with the findings from a recently updated meta-analysis of published, randomised clinical trials in patients with cancer receiving chemotherapy by the Cochrane Collaboration (Bohlius *et al*, 2006). A shift of the overall HR for mortality towards a more favourable outcome for patients in the control group compared to those receiving ESA treatment is different from the results of an earlier meta-analysis by the same group where a trend towards increased survival in patients treated with ESAs was shown (Bohlius *et al*, 2005). However, as in the present updated meta-analysis of controlled clinical trials with epoetin-*β*, the results of the updated meta-analysis seem to be driven by inclusion of data from studies, which allowed enrollment of patients with a baseline Hb up to and above 13 g dl^−1^. The outcomes reported in these trials have been either negative (Henke *et al*, 2003, Leyland-Jones *et al*, 2005) or neutral (Aapro *et al*, 2008; Strauss *et al*, 2008). An Hb initiation level above 11 g dl^−1^ is not in line with the current EORTC treatment recommendations (Bokemeyer *et al*, 2007). The subgroup analyses in our updated meta-analysis for epoetin-*β* are fully supportive of this conclusion.

To date, the results of two prospective, randomised studies suggesting that ESA treatment may have a negative impact on survival have been published in detail (Henke *et al*, 2003; Leyland-Jones *et al*, 2005). A negative impact of ESA treatment on survival is also suggested by three recent studies (Overgaard *et al*, 2007; Wright *et al*, 2007; Smith *et al*, 2008), two of which (Overgaard *et al*, 2007; Smith *et al*, 2008) have not yet been reported in full. The findings of Henke *et al* (2003) and Leyland-Jones *et al* (2005) should, however, be interpreted with caution as a number of limitations associated with the studies have been identified including baseline imbalances in prognostic factors, which favoured the placebo arm in both (Dunst 2004; Leyland-Jones and Mahmud, 2004; Vaupel and Mayer, 2004). Similar caution has to be exercised with respect to the interpretation of the other three studies mentioned above as they were either not designed to assess survival, have been terminated early, with a very limited sample size (Wright *et al*, 2007), have not been fully reported (Overgaard *et al*, 2007) or are in advanced patients not receiving chemotherapy (Smith *et al*, 2008) and do therefore not allow a conclusive interpretation. A common feature of these five studies is that all were conducted outside the currently approved indications and all were performed in predominantly mild or nonanaemic patients, with target Hb levels higher than those recommended by the EORTC guidelines (Bokemeyer *et al*, 2007), for the use of ESA therapy in cancer patients treated with chemotherapy.

### Tumour progression

The results in this updated meta-analysis do not suggest an increased risk for disease progression in patients receiving epoetin-*β* treatment *vs* those receiving standard care, but show the risk to be similar, with a trend towards a reduction in risk favouring patients receiving epoetin-*β*. When this analysis was restricted to patients with Hb intervention levels of ⩽11 g dl^−1^ in line with recent EORTC guidelines (Bokemeyer *et al*, 2007), the results showed a statistically significantly lower risk for disease progression in patients receiving epoetin-*β*. A similar finding of a more favourable outcome with respect to tumour progression in patients treated with ESA *vs* control was recently reported by the Cochrane Collaboration (Bohlius *et al*, 2006), as well as in a systematic review of 46 ESA trials conducted for the National Institute of Clinical Excellence (NICE) (Wilson *et al*, 2007). Whether the obvious discrepancy between outcomes of disease progression favouring epoetin treatment and survival outcomes favouring control/placebo treatment may be caused by an underdiagnosis of fatal TEEs (see section below) must remain speculative.

### Thromboembolic events

The present analysis showed a significantly increased TEEs rate with epoetin-*β* compared with control (7 *vs* 4%; *P*=0.008). These results are consistent with those reported in both meta-analyses of the Cochrane Collaboration (Bohlius *et al*, 2005, 2006). The risk of TEEs was shown to be higher in the subgroup of patients with solid tumours compared with those with nonmyeloid haematological malignancies. This may be driven mainly by the differences in TEEs risk in the underlying cancer population due to disease stage and activation of the coagulation system. Despite the well-known increased incidence of TEEs associated with epoetin-*β* treatment in the present analysis, importantly, the incidence of TEEs-related mortality was similar between the two treatment groups (1% in each group).

### CONCLUSIONS

The results of this meta-analysis including all prospective, randomised studies conducted with epoetin-*β* in cancer patients showed no evidence for a significantly negative effect of epoetin-*β* treatment on survival in patients with metastatic cancer. Furthermore, there was no negative effect of epoetin-*β* on tumour progression. The risk of TEEs was consistent with the increased TEEs risk observed within the ESA class in general, with a higher incidence of TEEs in patients with solid tumours. Predefined subgroup analyses in patients with an initiation Hb level corresponding to the current EORTC treatment guidelines (i.e., Hb⩽11 g dl^−1^) confirm the safety of epoetin-*β* in the treatment of anaemia in patients with metastatic cancers receiving concurrent chemotherapy when used within its licensed indication.

## Figures and Tables

**Table 1:** Main features of randomised clinical trials of epoetin-*β* in patients with cancer

**Study**	**Design and no. of patients (epoetin-*β*/control)**	**Diagnosis**	**Epoetin-*β* dosage and duration of therapy**	**Control**	**Cancer treatment**
ten Bokkel Huinink *et al* (1998) (MF4249)	o, pg *n*=83/87	Ovarian cancer, Hb<13 g dl^−1^	150 or 300 IU kg^−1^ 3 × week × 6 months	Standard therapy	Chemotherapy
Österborg *et al* (1996) (MF4250)	o, pg *n*=*95/49*	MM, NHL, CLL; transfusion-dependent, Hb<10 g dl^−1^	2000–10 000 IU day^−1^ titrated or 10 000 IU day^−1^ fixed dosage × 24 weeks	Standard therapy	Chemotherapy
Rau *et al* (1998) (MF4252)	db, pc and pg *n*=*28/26*	Resectable rectal cancer, Hb⩾12.5 g dl^−1^ (men); ⩾12 g dl^−1^ (women)	200 IU kg^−1^ daily × 11 days	Placebo	Surgery
Kettelhack *et al* (1998) (MF4253)	db, pc *n*=*52/57*	Colorectal cancer suitable for hemicolectomy, Hb>8.5–13.5 g dl^−1^	20 000 IU day^−1^ × 10–15 days	Placebo	Surgery
Data on file (Study MF4266)	o, pg *n*=*10/10*	AML	10 000 IU day^−1^, then weekly or twice weekly × ⩽30 weeks	Standard therapy	Chemotherapy
Cazzola *et al* (1995) (MF4313)	o, pg *n*=*117/29*	MM, NHL, CLL; transfusion-independent, Hb⩽11 g dl^−1^	1000, 2000, 5000 or 10 000 IU day^−1^ × 8 weeks	Standard therapy	Chemotherapy
Oberhoff *et al* (1998) (MF4421)	pg, *n*=*114/104*	Solid organ tumours, Hb⩽11 g dl^−1^	5000 IU day^−1^ × 12–24 weeks	Standard therapy	Chemotherapy
Boogaerts *et al* (2003) (MF4321)	o, pg *n*=*131/128*	Malignant disease, Hb⩽11 g dl^−1^	150 IU kg^−1^ 3 × week adjusted for Hb response × 12 weeks	Standard therapy	Chemotherapy
Österborg *et al* (2002); Österborg *et al* (2005) (MF4467)	pc, db and pg *n*=*170/173*	MM, NHL, CLL; transfusion-dependent and epo-deficient, Hb⩽10 g dl^−1^	150 IU kg^−1^ 3 × week adjusted for Hb response × 16 weeks, 12-month study period^*^	Placebo	Chemotherapy
Henke *et al* (2003) (MF4449)	pc, db and pg *n*=*171/180*	Head and neck cancer, Hb<13 g dl^−1^ (men), <12 g dl^−1^ (women)	300 IU kg^−1^ 3 × week, 6–8 weeks, 60-month study period	Placebo	Radiotherapy
Strauss *et al* (2008) (MO16375)	o, pg *n*=*34/40*	Cervical cancer Stage FIGO IIB-IVA, Hb 9–13 g dl^−1^	150 IU kg^−1^ 3 × week, 8–14 weeks, 6-month study period	Standard therapy	Radio-chemotherapy
Aapro *et al* (2008) (BA16756)	o, pg *n*=*231/232*	Breast cancer, Hb <12.9 g dl^−1^	30 000 IU weekly × 24 weeks, 24-month study period	Standard therapy	Chemotherapy

Abbreviations: AML=acute myeloid leukaemia; CLL=chronic lymphocytic leukaemia; db=double-blind; Hb=haemoglobin; MM=multiple myeloma; NHL=non-Hodgkin's lymphoma; o=open design; pc=placebo-controlled; pg=parallel group. Patients had anaemia unless stated otherwise, and standard therapy consisted of antitumour treatment plus blood transfusion as required.

^*^Information on disease progression not collected during the follow-up period of this study.

**Table 2:** Baseline characteristics of pooled study populations

**Parameter**	**Control (*N*=1053)**	**Epoetin-*β*** **(*N*=1244)**
*Gender* (% *male/female*)	37*/*63	38*/*62
		
*Race*		
*n*	921	1069
Caucasian	882 (96%)	1029 (96%)
Other	39 (4%)	40 (4%)
*Mean age in years (range)*	58.8 (19–91)	59.3 (20–87)
*Mean weight in kg (range)*	67.7 (30.0–131.5)	67.1 (35.0–118.0)
*n*	1048	1235
		
*Mean height in cm (range)*	166.7 (140–198)	166.4 (126–198)
*n*	809	1012
		
*Tumour type, n* (%)		
*Haematological*	331 (31.4)	465 (37.4)
Acute myeloid leukaemia	10 (3.0)	10 (2.2)
Multiple myeloma	125 (37.8)	204 (43.9)
Non-Hodgkin's lymphoma	195 (58.9)	247 (53.1)
Hodgkin's lymphoma	1 (<1)	4 (<1)
		
*Solid*	722 (68.6)	779 (62.6)
Breast	261 (36.2)	
Head/neck	174 (24.1)	261 (33.5)
Gynaecological	133 (18.4)	181 (23.2)
Gastrointestinal	96 (13.3)	186 (23.9)
Other	58 (8.0)	100 (12.8)
		51 (6.6)
*Haemoglobin*		
N	1050	1241
Mean (range)	10.6 (5.7–16.7)	10.5 (4.2–17.1)
Median	10.5	10.4

Data were collected from all 2297 patients unless otherwise stated.

**Table 3:** Kaplan–Meier and Cox regression analysis of survival and time-to-event data

	**Control (*N*=1053)**			**Epoetin-*β* (*N*=1244)**				
**Patient group**	**Total events**	**Mean patient-years of follow-up**	**Events per patient-year**	**Total events**	**Mean patient-years of follow-up**	**Events per patient-year**	**Hazard ratio (95% CI)**	***P*-value^a^**
*Pooled population of 12 controlled studies*								
*Overall survival*								
Total	99	0.32	0.29	136	0.33	0.33	1.13 (0.87–1.46)	0.355
Solid	58	0.32	0.25	78	0.34	0.30	1.17 (0.83–1.64)	
Non-myeloid haematological	41	0.34	0.37	58	0.32	0.39	1.04 (0.69–1.55)	
								
*Time to progression*								
Total	254	0.28	0.86	268	0.29	0.74	0.85 (0.72–1.01)	0.072
Solid	171	0.27	0.88	173	0.29	0.76	0.85 (0.68–1.05)	
Non-myeloid haematological	82	0.31	0.81	93	0.29	0.69	0.84 (0.62–1.13)	
								
*Time to thromboembolic event*								
Total	46	0.32	0.14	88	0.32	0.22	1.62 (1.13–2.31)	0.008
Solid	29	0.32	0.13	61	0.33	0.24	1.92 (1.24–2.99)	
Non-myeloid haematological	17	0.34	0.15	27	0.32	0.18	1.18 (0.64–2.16)	
								
*Pooled population of studies with long-term follow-up*								
*Overall survival*								
Total	371	1.54	0.39	396	1.45	0.44	1.13 (0.98–1.31)	0.082
Solid	262	1.61	0.37	286	1.49	0.43	1.17 (0.99–1.39)	
Non-myeloid haematological	109	1.37	0.46	110	1.36	0.48	1.04 (0.80–1.36)	
*Time to progression*								
Total^b^	260	1.10	0.54	270	0.98	0.62	1.13 (0.95–1.34)	0.165

a Log-rank test *P*-value epoetin-*β* *vs* placebo/control.

b All studies were in patients with solid tumours; ‘events’ refers to number of deaths for ‘overall survival’, number of malignant disease progressions for ‘time to progression’ and number of thromboembolic events for ‘time to thromboembolic event’.
